# State of the Art Biocompatible Gold Nanoparticles for Cancer Theragnosis

**DOI:** 10.3390/pharmaceutics12080701

**Published:** 2020-07-25

**Authors:** Moon Sung Kang, So Yun Lee, Ki Su Kim, Dong-Wook Han

**Affiliations:** 1 Department of Cogno-Mechatronics Engineering, College of Nanoscience and Nanotechnology, Pusan National University, 2 Busandaehak-ro 63 beon-gil, Geumjeong-gu, Busan 46241, Korea; mskang7909@gmail.com; 2 Department of Organic Materials Science and Engineering, College of Engineering, Pusan National University, 2 Busandaehak-ro 63 beon-gil, Geumjeong-gu, Busan 46241, Korea; nanobiosyl@gmail.com

**Keywords:** gold nanoparticle (AuNP), cancer therapy, theragnosis, photothermal treatment (PTT), photodynamic therapy (PDT), cancer imaging

## Abstract

Research on cancer theragnosis with gold nanoparticles (AuNPs) has rapidly increased, as AuNPs have many useful characteristics for various biomedical applications, such as biocompatibility, tunable optical properties, enhanced permeability and retention (EPR), localized surface plasmon resonance (LSPR), photothermal properties, and surface enhanced Raman scattering (SERS). AuNPs have been widely utilized in cancer theragnosis, including phototherapy and photoimaging, owing to their enhanced solubility, stability, biofunctionality, cancer targetability, and biocompatibility. In this review, specific characteristics and recent modifications of AuNPs over the past decade are discussed, as well as their application in cancer theragnostics and future perspectives. In the future, AuNP-based cancer theragnosis is expected to facilitate the development of innovative and novel strategies for cancer therapy.

## 1. Introduction

Gold, one of the historically precious materials of everyday human life, is utilized as currency, jewelry, accessories, and valuables, as well as in industries owing to its distinct characteristics. For example, gold is used in semiconductors because of its superior electrical conductivity and processability, while it is used in chemical processes as a catalyst [1,2,3,4]. Moreover, biomedical applications of gold have a long history. As early as 700 BC, in Etruria, gold was used in dental treatment [5]. The use of gold in ancient times for in vivo transplantations can be attributed to its extraordinary physicochemical properties. Gold can be molded into a desirable shape, has no toxicity to the human body, does not rust, and has no undesirable taste. The rapid development of nanotechnology has enabled the manufacture of gold nanoparticles (AuNPs) in different sizes and shapes, leading to their expanded and diversified use in many fields [6,7,8,9].

In particular, many studies have focused on utilizing AuNPs as cancer theragnostic probes, owing to their unique optical characteristics that are not observed in bulk gold formulations. For example, their scattering property, absorption/emission spectra, and photoluminescence property can change significantly size-dependently. Thus, AuNPs have tremendous potential for use as controllable fluorescent, non-bleaching imaging probes or contrast agents for bioimaging [10,11]. Furthermore, the photothermal properties of AuNP, such as its localized surface plasmon resonance (LSPR) which is caused by a collective oscillation of electrons at the surface, renders it useful as a therapeutic probe for photodynamic treatment (PDT) and photothermal treatment (PTT) [12,13]. Moreover, the superior biocompatibility and non-immunogenicity of AuNPs augment their potential for clinical use as cancer theragnosis probes.

**Figure 1:**
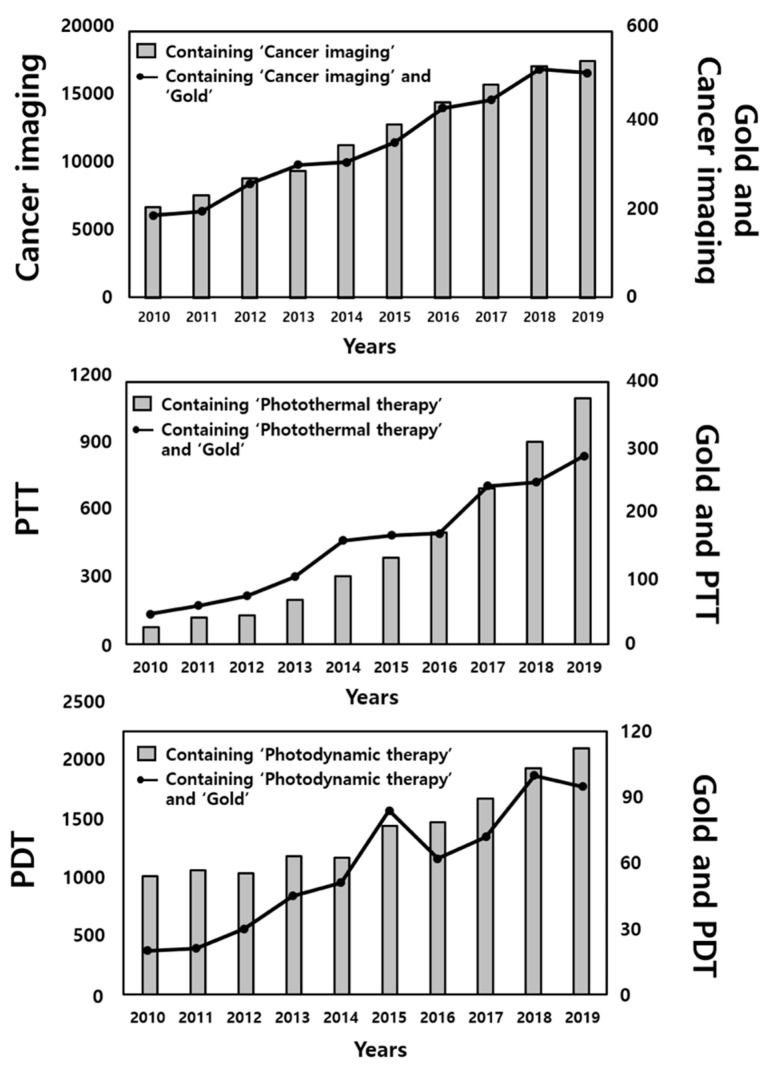
Numbers of recent publications featuring the terms gold and cancer imaging, photothermal treatment (PTT), or photodynamic therapy (PDT). Y-axes of graphs represent the numbers of published papers containing each of the indicated words. All values were obtained from PubMed.

Because research on cancer imaging, PTT, and PDT have increased in recent years, interest in investigating the use of gold in these applications has correspondingly increased by more than two-, seven-, and five-fold, respectively (Figure 1). In this review, we highlight the specific properties and biological features of AuNPs that have enabled their application in cancer theragnosis, with a focus on recent methods for their applications and future perspectives in cancer imaging and phototherapy, classified according to the synthesis methods and formulations of AuNPs (Table 1).

## 2. Key Properties of Gold Nanoparticles (AuNPs) Important for Cancer Theragnosis

### 2.1. Biocompatibility

Nanomaterials (NMs) for body transplants and injections should have biocompatible and bioinert properties. Since NMs can interact with mammalian cells and tissues in ways different from those of their bulk counterparts, their potential toxicity is still controversial. The toxicity of metals in the body is mostly due to their capacity to form metal cations that damage cell membrane. However, gold is relatively stable in the body because of its high reduction potential and is less likely to ionize than other metals. Previous studies have verified that gold is relatively safe both in vitro and in vivo, although the size and concentration of AuNPs should be within the recommended ranges [47,48,49,50]. Due to the nature of inorganic NMs, their decomposition and release in the body could be potentially hindered [51]. In addition, little is known about the processing and degradation of AuNPs after uptake by cells [52]. Murphy et al. emphasized the potential toxicity of AuNP [53] by demonstrating that a 13 nm citrate-capped AuNP showed non-cytotoxicity and promoted formation of abnormal actin filaments which led to decreased cell behavior. Meanwhile, toxicity can be different within a cell type or species; a 33 nm citrate-capped AuNP was found to be nontoxic to hamster kidney and human hepatocellular liver carcinoma cells, but cytotoxic to human carcinoma lung cell line. Therefore, more studies are required that investigate both the mechanism of action of the metabolic process of AuNPs and strategies that could enhance their biodegradation and release such as those that capitalize on surface chemistry, shape modulation, and size control [54].

Surface engineering of AuNPs has been reported as a good strategy for enhancing biocompatibility and functionality through interaction with cells or tissues. Surface ligands determine the physicochemical properties of AuNP surface, such as hydrophilicity, zeta potential, and dispersion in solution. Surface properties determine interactions among cells and AuNPs, cell membrane binding and permeability, immune reaction, and in vivo localization [55]. Appropriate surface engineering of AuNPs can markedly improve their biocompatibility and responsiveness to cells. Surface engineering of AuNPs is generally processed by functionalization with various moieties such as polymers, surfactants, proteins, peptides, or other ligands [56,57,58,59,60]. One example introduced by Liu et al. synthesized multidentate zwitterionic chitosan oligosaccharide (CSO) modified AuNPs by surface engineering [61]. The zwitterionic CSO was prepared by conjugating 2-acryloyloxyethyl phosphorylcholine (APC) to CSO with an amino group by a Michael addition reaction and further conjugated to lipoic acid (LA) by reacting the carboxylic acid group with the amine group using carbodiimide chemistry. The resultant LA-CSO-PC exhibited hemocompatibility, high cell viability, and cell membrane integrity at concentrations ≤0.12 mM, suggesting that surface engineering can facilitate the preparation of AuNPs as biomedicine.

The size and shape of NPs are crucial factors affecting biocompatibility; they are dramatically changed by endocytosis, protein absorption, gene regulation, and physical damage to membrane integrity [62]. For example, Vácha et al. demonstrated that passive endocytosis varied depending on the size and shape of the NPs. Differential membrane-binding strengths were observed, and spherocylindrical-shaped NPs were found to be more efficiently endocytosed than sphere-shaped NPs; moreover, the transport of NPs with sharp edges was suppressed [63]. Vecchio et al. quantitatively compared size-dependent toxicity of citrate-capped AuNPs and found that, as the particle size decreased, the cell viability decreased and the amount of reactive oxygen species increased; this relationship was especially proportional within a size range of 5 to 80 nm [64]. Sangabathuni et al. showed that AuNPs, depending on their shape, had different toxicity, biodistribution, and sequestration, in Zebrafish [65], and that nanorods uptake and clearance were faster, but nanostars decomposition was slower and stayed longer in the body.

### 2.2. Physicochemical Characteristics

#### 2.2.1. Enhanced Permeability and Retention (EPR) Effect

The EPR effect is a phenomenon in which liposomes, macromolecular drugs, and NPs accumulate in cancer tissues for a longer period of time than in normal tissues. It is known that 200 nm–1.2 µm sized particles tend to accumulate more in tumors, depending on the tumor type; 200 nm is often considered to be a favorable size for the EPR effect [66,67,68]. The EPR effect is induced by certain characteristics of tumors, such as aberrant vasculature with extensive production of vascular permeability factors that stimulate extravasation and lack of lymphatic drainage, and wider lumen between tissues [69]. Consequently, injected AuNPs can remain longer and are tumor-specifically retained; thus, they can be applied as a passive tumor targeting strategy. Many studies have reported that the AuNPs without any tumor-targeting domain are relatively more abundant in cancer tissues, owing to the EPR effect [70,71,72].

#### 2.2.2. Localized Surface Plasmon Resonance (LSPR)

SPR (surface plasmon resonance) is defined as the oscillation of electron on a conductive metal surface that forms an interface with a non-conductive substrate. SPR leads to a unique optical phenomenon in which light is scattered or absorbed when a conductive metal is irradiated with light of a specific wavelength that matches the plasmon oscillation cycle. Although most metallic materials absorb light in the ultraviolet region, Au absorbs light in the visible region, facilitating wider application in optical imaging. LSPR is one of the unique characteristics of nanomaterial. Unlike bulk Au, the surface size of an AuNP is very small and its plasmons do not move; instead, the plasmons oscillate collectively with a specific wavelength at the NP surface, which is variable depending on the particle size and formulation. Because the absorption and scattering of light by AuNPs depend on their size and formulation, they are widely applied as contrast agents in X-ray imaging, computed tomography (CT), photoacoustic tomography (PAT), and fluorescence imaging in vivo and in vitro [73,74,75,76,77,78].

#### 2.2.3. Photothermal Effect

Accurate temperature control is a key factor for successful photothermal treatment of cancer. Therefore, understanding the photothermal properties of AuNPs is important. The photothermal effect is caused by photon irradiation and conversion of absorbed energy to thermal energy. Localized heating can result in the elimination of a targeted tissue; therefore, it can be applied as a noninvasive therapy. In addition, the generated heat is confined around the particles, enabling localized thermal toxicity to targeted cancer tissues. Basically, the light absorbed by AuNPs is converted to heat to induce a photothermal effect. In particular, the photothermal effect of AuNPs is strengthened by LSPR, as the oscillating excited electrons scatter each other. The quantized collective and coherent oscillation of the conduction band electrons is driven by light when resonance condition matches, and then the photothermal effect of the AuNP is significantly enhanced. The photothermal effect by LSPR enhances the localization of heat because, although the released energy has high intensity, the total capacity is small [79].

Among Au-based nanomaterials, nanospheres have an absorbance peak near 520 nm and do not absorb strongly in the near-infrared (NIR) range well, meanwhile, nanorods or nanoshells can strongly absorb NIR light for electron oscillation allowing deeper penetration of tissues. Therefore, in vivo imaging using Au nanorods or Au nanoshells is more feasible than AuNPs. However, nanorods and nanoshells are rarely fabricated as small size to be cleared from the body [80]. A possible solution could be rod-shaped assembly of AuNPs that could absorb NIR light efficiently by plasmon coupling of individual AuNPs [81]. Taken together, AuNPs hold a particular interest in the photothermal effect owing to their extraordinary photon-to-thermal energy conversion efficiency under NIR irradiation [82,83].

#### 2.2.4. Surface Enhanced Raman Scattering (SERS)

The typical Raman cross-section is 10^−30^ cm^−2^ sr^−1^, which is 10^14^ times smaller than the average cross-section of fluorescent dye (10^−16^ cm^−2^ sr^−1^). It has the limitations of slow acquisition speed and difficult applicability in biological imaging [84]. However, when a localized surface plasmon of metal nanoparticles has a specific resonance condition (when the frequency of incident light and the frequency of collective motion of the conduction band electrons accord), the surface plasmon generates an induced electromagnetic field. Therefore, when AuNP is irradiated with light of a specific wavelength, the electromagnetic field on its surface is amplified such that the Raman signal is amplified when an organic substance attaches to the AuNP surface. Studies have confirmed that the SERS effect could amplify Raman cross-section to a degree similar to that of a fluorescent dye [85,86,87]. SERS Raman spectroscopy has achieved high resolution comparable to that of fluorescence microscopy and has potential for application in spectroscopic cancer diagnosis [88,89,90].

## 3. Phototherapy

### 3.1. Photothermal Therapy (PTT) with Gold Nanoparticles

AuNPs have attracted the attention of researchers for their application in photothermal cancer therapy owing to their surface resonance (SPR) characteristics resulting from energy excitation that can be achieved by irradiating the specific wavelength light to the AuNPs. LSPR due to laser irradiation causes enhanced absorption by gold nanoparticles, resulting in photothermal heating that can be used to damage cancer cells, bacteria, and viruses [91,92,93]. Recently, various types of AuNPs, such as gold nanospheres [94,95,96,97], gold nanorods [14,15,16,17,98,99,100,101,102,103,104], gold nanoshells [18,19,105,106,107,108,109], gold nanocages [30], gold nanostars [31], gold nanoflowers [20,21], and gold nanoring [71] have been investigated both in vitro and in vivo, because the absorption/scattering ratio could be controlled by adjusting the size, morphology, and surface properties of the NPs.

Xiaochao et al., in 2012, reported that gold showed little cytotoxicity in cells at a relatively low concentration of AuNPs and short incubation time. In this study, it was found that laser irradiation could induce killing of gold-targeted L-428 cells with high efficiency due to the photothermal effect of the AuNPs [97]. Gold nanorods have been actively studied due to their improved photothermal effects on cancer cells and are used in certain applications because of their higher absorption cross-sections at near-infrared frequencies per unit as compared with other AuNPs [104]. In 2016, the improved photothermal efficiency of gold nanorods that transported macrophage vehicles in vivo was demonstrated by Li and coworkers, who showed that BSA-coated Au nanorods-laden macrophages significantly improved photothermal conversion almost throughout the tumor and minimized tumor recurrence rate as compared with free BSA-coated Au nanorods [19]. In 2014, Popp et al. used an NIR light emitting diode (LED) as a light source and implemented gold nanorods mediated photothermal therapy in vitro and in vivo; they found that the LED light source effectively and quickly heated the in vitro and in vivo models when combined with gold nanorods. In that study, the volume of the tumor decreased and the animal survival rate increased when gold nanorods were used as compared with treatment using melanoma drugs [15]. Du et al., in 2015, prepared a core-shell composite consisting of polypyrrole-stabilized gold nanorods with two-photon photothermal efficiency and good photostability by facile interfacial polymerization. The resulting core-shell composite showed high efficiency in inhibiting the proliferation of tumor cells, while minimizing photothermal damage to the normal tissues [16]. Furthermore, in 2015, Sugiura and coworkers proposed a temperature control system that allowed metastatic lymph nodes within or outside the area accessible for surgical dissection to be treated by photothermal therapy using NIR laser light and gold nanorods with controlled surface cooling [17]. Therein, the high temperature zone and the lymph node site where damage was observed were localized to areas up to 3 mm in depth.

Nanoshells consisting of a dielectric core with a thin gold shell of thickness measuring a few nanometers show surface plasmon resonance peaks in the NIR region [108,109]. In 2011, Carpin et al. used immunoconjugated gold nanoshells, fabricated by adding anti-HER2 to the gold surface of the nanoshells, as a targeting modality for photothermal therapy and demonstrated the successful targeting and ablation of trastuzumab-resistant cells using anti-HER2-conjugated silica-gold nanoshells and a near-infrared laser [18]. In 2011, Kennedy reported two trastuzumab-resistant breast cancer cell lines, BT474 AZ LR and JIMT-1, in which photothermal therapy based on gold nanoshells showed the possibility for therapy-resistant breast cancer resection [19]. In addition, Chhetri et al. showed that the photothermal conversion of gold-silica nanoshells was much higher than that of gold nanorods in hybrid murine macrophages employed to transport nanoparticles into human glioma spheroids [22,110].

A cancer treatment strategy using gold nanocages that have the advantages of smaller size, higher specific surface area, and strong, tunable absorption in the NIR transparent window, was investigated by Gao et al., in 2012. They fabricated hypocrellin-loaded gold nanocages with high two-photon efficiency and demonstrated that photodynamic anticancer treatment was significantly improved by photothermal effect under two-photon illumination due to internalization of the nanocomplex by cancer cells [30]. In 2015, Huang et al. proposed γFe_2_O_3_@Au magnetic gold nanoflower-mediated NIR photothermal cancer theragnostics. The γFe_2_O_3_@Au magnetic gold nanoflowers exhibited good spatial resolution in MR imaging for precise tumor localization and high-resolution photo acoustics imaging, and efficiently ablated tumors under NIR irradiation [20].

**Figure 2:**
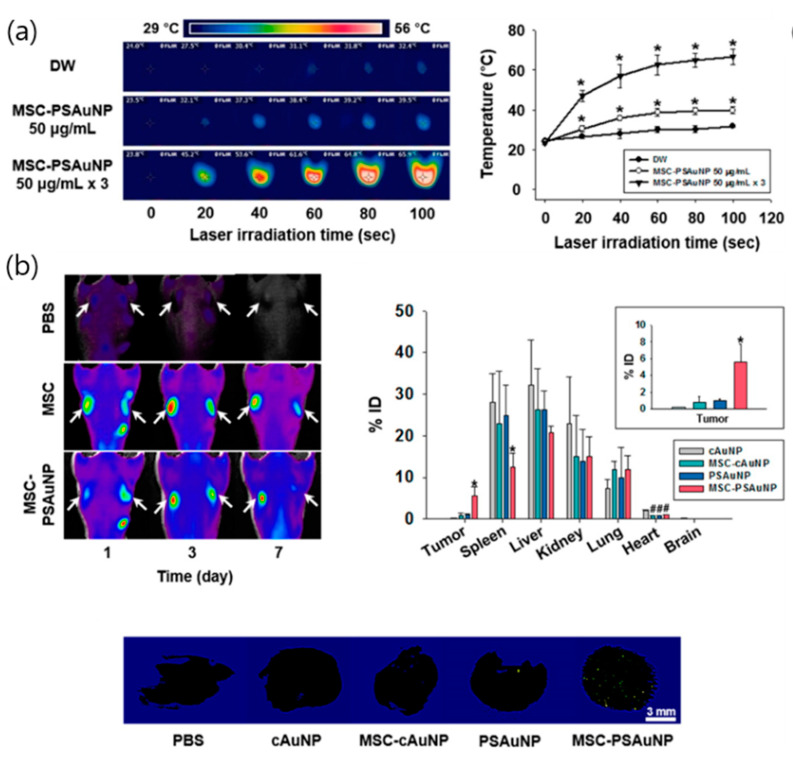

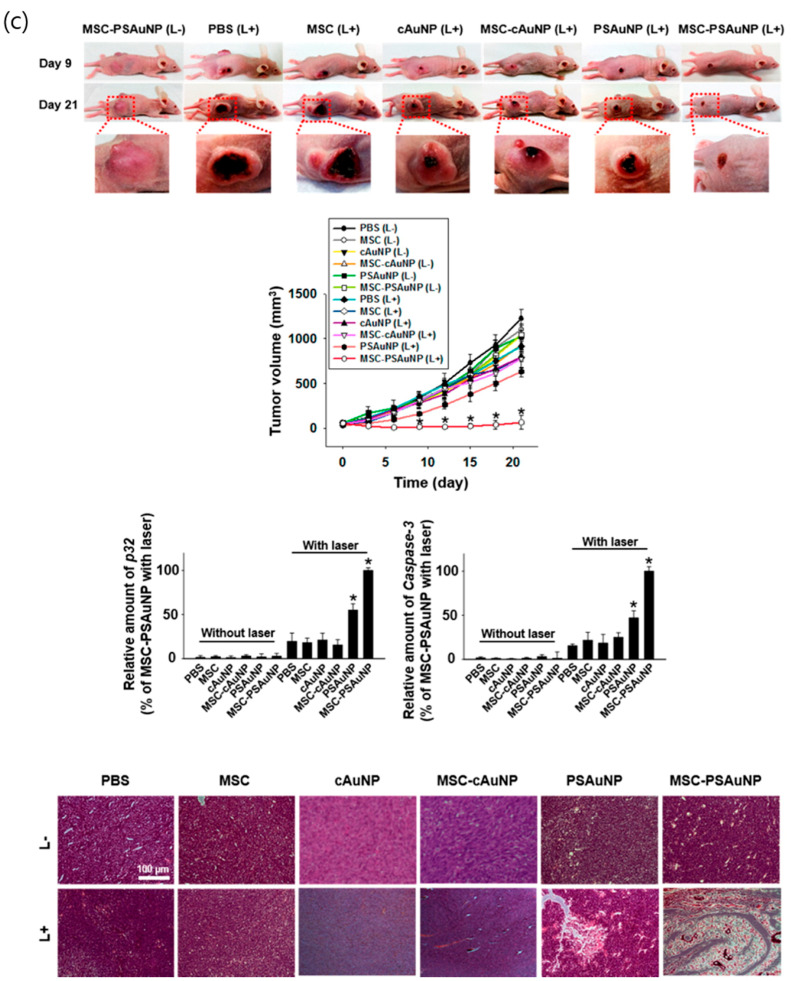
(**a**) Temperature measurements during laser irradiation to demonstrate photothermal effects of a single treatment and three consecutive treatments of mesenchymal stem cells (MSCs) with PSAuNPs, * *p* < 0.05 versus any group, ^#^ *p* < 0.05 versus MSC-cAuNP 90 μg/mL group; (**b**) In vivo tumor targeting and biodistributions of the AuNPs, * *p* < 0.05 versus any other group. ^#^ *p* < 0.05 versus the cAuNP group; (**c**) Volume profiles of each tumor tissue; apoptotic activity in the tumor tissues after laser irradiation and hematoxylin and eosin staining images of thin sections of the tumor tissues with or without irradiation, * *p* < 0.05 versus any other group. Reproduced from [111], copyright permission by ACS Publication 2015.

Recently, a strategy that utilized a synergy effect and combined the photothermal effect of AuNPs and the tumor-inducing effects of stem cells was studied for cancer targeting. In 2015, Kang et al. demonstrated that pH-sensitive AuNPs (PS-AuNPs) could aggregate in the mildly acidic endosomes of mesenchymal stem cells (MSCs) and used them as vectors for photothermal therapy [111]. The resulting aggregated structures showed a higher cellular retention, which was important for the cell-based delivery system as compared with pH-insensitive gold nanoparticles. Furthermore, the photothermal effects of PS-AuNPs were enhanced three times in multiple treatments of MSCs with PS-AuNPs as compared with a single treatment of the same capacity. Moreover, injection of MSCs with PS-AuNPs accumulated higher amounts and also showed an even distribution of AuNP in tumor tissues as compared with the other groups. In vivo, MSCs with PS-AuNP exhibited high efficiency for targeting tumor tissues, improved photothermal efficiency, and increased anticancer therapeutic efficacy under NIR irradiation (Figure 2).

### 3.2. Photodynamic Therapy (PDT) of Gold Nanoparticles

Photodynamic therapy is another promising treatment method for cancers. PDT uses oxygen from tissues, light, and photosensitizers (PS) that can be excited by light of specific wavelengths to generate reactive oxygen species (ROS) via energy transfer, which leads to cell death by apoptosis [112,113]. While PTT is independent, PDT is dependent entirely on the oxidative nature of ROS [114]. Therefore, the role of PS is very important in PDT; if a suitable PS is chosen, PDT has the advantages of good tolerance and repeated use at the same site and is a minimally invasive procedure [115,116]. AuNPs are good biocompatible carriers of PS and can encapsulate PS or combine them to surfaces [117,118,119]. Many researchers are working to develop improved PS for enhancing the efficiency of PDT and it has been reported that AuNPs can increase the singlet oxygen generation (SOG) of various PS and improve PDT efficiency [120].

The generation of cytotoxic single oxygen by phthalocyanine stabilized gold nanoparticles was first demonstrated by Hone et al. [121], who prepared photosensitizer phthalocyanine functionalized gold nanoparticles combined with a tetraoctylammonium bromide (TOAB) phase transfer reagent and showed that the SOG of the composites (photosensitizer/gold/phase transfer reagent) was higher than that of the free photosensitizer. Since then, the use of AuNPs for photodynamic therapy has been investigated by many groups [122,123]. In 2010, Camerin et al. reported the efficiency of a Zn(II)-phthalocyanine disulphide (C11Pc)-nanoparticle conjugate as a photodynamic therapy agent in C57 mice bearing a subcutaneously transplanted amelanotic melanoma. The fabricated AuNP-bound C11Pc showed a more selective response to the cancer tissue as compared with free C11Pc and the findings suggested that the photodynamic therapy promoted by C11Pc mainly acted via vascular damage [23]. However, without an apparent decrease of PS, the conjugates remained in the liver and spleen, showing prolonged persistence in the liver. To overcome this, subsequently, Russell et al. fabricated PEGylated AuNP-C11Pc conjugates that attached anti-HER2 monoclonal antibodies to a PEG chain for targeted delivery to breast cancer cells. Although in vivo analyses were not presented, the efficacy and selective targeting of the conjugates in PDT were demonstrated [24]. In 2015, Savarimuthu et al. reported that cancer cells were targeted using folic acid as a mark; however, only in vitro studies were performed [25].

In an in vitro comparison of the efficiency of photodynamic therapy with hematoporphyrin-gold nanocomposites of different diameters, in 2010, Gamaleia et al. fabricated a conjugate consisting of hematoporphyrin and AuNPs and demonstrated that larger particles performed better because they could transfer more hematoporphyrin to malignant cells [26]. In 2012, Khaing Oo et al. demonstrated that a localized electromagnetic field, attributed directly to the AuNP-enhanced and size-dependent generation of ROS from protoporphyrin IX (PpIX), was a result of the SPR of the AuNPs upon light irradiation [27]. Similarly, Zhang et al., in 2015, showed that PpIX-AuNPs conjugates improved SOG by about 2.5-fold as compared with free PpIX. In their study, local field enhancement increased with an increase in the AuNP size and they found that this enhancing effect of SOG was dependent not only on the size but also on the wavelength of light [28].

The studies reported so far have shown that both photothermal and photodynamic therapies are very important and effective in the treatment of cancer. Therefore, strategies that combine these therapies are expected to have more efficient and powerful cancer cell damaging effects. In 2010, Kuo et al. were the first to demonstrate that AuNPs could be used simultaneously as contrast agents in imaging, as well as in PTT and PDT, to image and more efficiently kill malignant A549 cells as compared with PTT or PDT alone [29]. Subsequently, in 2012, the group investigated dual-modality PTT and PDT using both AuNPs and gold nanorods conjugated with indocyanine green as PS. The results showed enhanced photodestruction, photostability, and efficient killing of cancer cells as compared with PTT and PDT alone [32].

**Figure 3:**
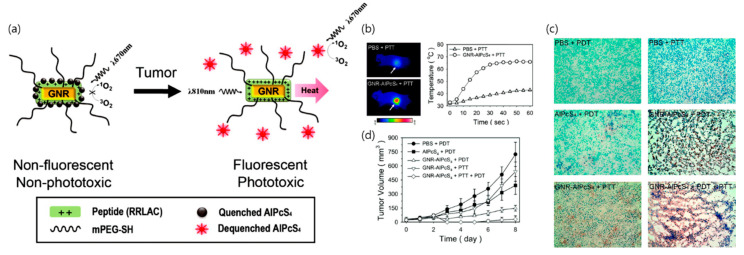
In vivo PTT and PDT. (**a**) The PEG-gold nanorod-RRLAC/Al(III) phthalocyanine chloride tetrasulfonic acid (GNR-AlPcS_4_) complex for NIR fluorescence imaging and tumor phototherapy; (**b**) Thermographic images captured after 1 min of light illumination, and thermographic monitoring in the tumors of GNR-AlPcS_4_-injected and PBS-injected mice; (**c**) TUNEL staining of the tissue sections (magnification × 20). Normal or apoptotic cell nuclei are shown in green and brown, respectively. Empty areas in the tissue sections (GNR-AlPcS_4_ complex + PDT and GNR-AlPcS_4_ complex + PTT + PDT) are due to washout of the destroyed tumor cells during the staining procedure; (**d**) Tumor size in each treatment group by the indicated days. Points, mean; bars, standard deviation. PBS + PDT (*n* = 7), free AlPcS_4_ + PDT (*n* = 7), GNR−AlPcS_4_ complex + PDT (*n* = 7), GNR-AlPcS_4_ complex + PTT (*n* = 5), GNR-AlPcS_4_ complex + PTT + PDT (*n* = 7), *n* = number of tumors involved. Reproduced from [33], copyright permission by ACS Publication 2011.

In 2011, Jang et al. proposed gold nanorod-PS complex for noninvasive NIR fluorescence imaging and cancer therapy [33]. Using in vivo NIR fluorescence imaging, the study clearly identified tumor sites as early as 1 h after intravenous injection of the complex and the tumor-to-background ratio increased with time. Furthermore, the dual strategy exhibited 95% decrease in tumor growth as compared with the 79% decrease observed in PDT alone (Figure 3). In 2014, Terentyuk et al. showed that dual-modality PTT and PDT gold nanorods/hematoporphyrin-loaded silica shell complex caused a dramatic decrease in tumor volume as compared with PDT plus PTT and PDT alone [34].

## 4. Photoimaging

### 4.1. Surface Enhanced Raman Spectroscopy (SERS)

**Figure 4:**
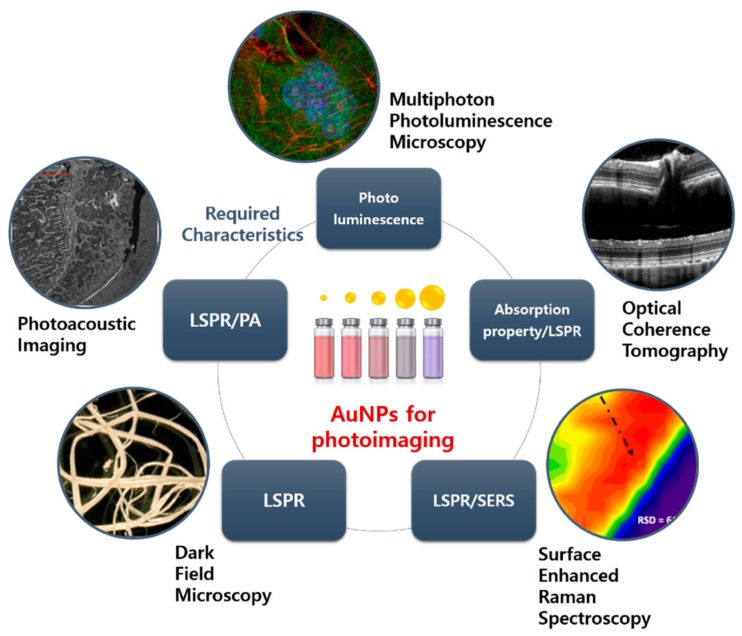
AuNP-based photoimaging. Each imaging system is arranged by required characteristics of AuNP. Image courtesy of Nikon (dark field microscopy), YSP (photoacoustic imaging (PAI)), phoenix (optical coherence tomography (OCT)), ZEISS (multiphoton photoluminescence microscopy), Sigma-Aldrich (surface enhanced Raman scattering (SERS)), respectively.

As described earlier, surface enhanced Raman spectroscopy is a diagnostic method involving signal amplification by conjugation of an organic substance reacting with a target on the AuNP surfaces. Cell imaging, such as in vivo tissue imaging or hematologic analysis, is a commonly used noninvasive cancer detection method. Fluorescence dyes are conventionally used to detect the exhibiting of specific surface receptors on cancer cells. However, the use of fluorescence dyes has several limitations. Imaging of deeper tissue (>500 µm) requires NIR light; however, there are relatively few suitable fluorescence dyes exhibiting absorption and emission at the NIR region [124]. Moreover, fluorescence dyes have a photobleaching effect and easily result in spectral overlap when different fluorophores are employed on multiple cell surface receptors. To overcome these disadvantages, SERS with AuNPs have emerged as a new detection tool with controllable spectral properties, high sensitivity with low sample concentration, and stability. Zhang et al. synthesized AuNP-based SERS probe for leukemic lymphocytes [35]. In their work, 4-mercaptobenzoic acid (MBA) was adsorbed on AuNP by its mercapto group (AuNP/MBA) and CD3 or CD19 antibody was immobilized on the surface of the AuNPs/MBA by electrostatic adsorption. Finally, PEG was added to the prepared probe to prevent aggregation and precipitation. The result indicated that two characteristic Raman signals of MBA were significantly enhanced (1076 and 1584 cm^−1^) relative to MBA mixture. Furthermore, the leukemic lymphocytes, Jurkat cells, and Raji cells were labelled by CD3 and CD19 conjugated AuNP/MBA, respectively, suggesting that the prepared AuNP/MBA enabled specific selectivity and targetability. Natalie et al. coated AuNP with porphyrin-phospholipid conjugate for structurally stable and biocompatible SERS probe [36]. The Mn-porphyrin-lipid conjugate was synthesized using NIR photosensitizer- pyropheophorbide-a, which was linked to a single acyl chain phospholipid-lysophosphatidylcholine at the glycerol backbone, and then coated with AuNPs in an aqueous solution. Validation by spectroscopy and confocal microscopy showed that the SERS probe was capable of cellular imaging with high cytocompatibility for HepG2 and A549 cells. Quynh et al. used 4-aminothiolphenol (4-ATP)-attached AuNPs to detect basal cell carcinoma (BCC) of the skin [37]. Au-4ATP was prepared by a wet chemical process and, for antibody conjugation, 1-ethyl-3-(3-dimethylaminopropyl) carbodiimide (EDC) was mixed with BerEP4, then added to the Au-4ATP solution. The enhanced Raman band corresponding to a C-S stretching vibration in 4-ATP was observed and conjugation with BerEP4 antibody did not change the frequency and intensity of the band. In a fingerprint method using this SERS, 40 × 40 µm skin images were obtained and clearly showed the position of the tumor site, enabling fast and selective diagnosis of BCC without subjective interpretation (Figure 4).

### 4.2. Multiphoton Photoluminescence Imaging

Multiphoton photoluminescence imaging is a microscopy technique that combines the optical techniques of laser scanning microscopy with long wavelength multiphoton fluorescence excitation to capture fluorophore-tagged specimen with high resolution. When the low-energy photonic excitation of electrons occurs from the occupied d band of AuNPs to the unoccupied sp band, a luminescent signal by hole-electron recombination follows, which is called photoluminescence. AuNPs have several advantages as photoluminescent imaging probes. The photoluminescence of bulk gold is negligible, however, significantly increased by enhancements of scattering or absorption cross-sections of nanoparticle plasmons. Moreover, AuNPs have superior processability and are relatively inert, as well as easily attached to other moieties [125]. Nagesha et al. demonstrated in vitro imaging of two different cell types (*Dictyostelium discoideum* and mouse embryonic stem cells) using multi-photon photoluminescence of AuNPs [126]. The uptake of AuNPs did not hinder stem cell proliferation and photoluminescence imaging was possible even after many passages, indicating their applicability in stem cell proliferation experiments. Lai et al. synthesized 11-mercaptoundecanoid acid (MUA) coated AuNP (MUA-AuNP) for multimodal biomedical imaging. [38]. The prepared probe showed intense visible photoluminescence and very high accumulation in cancer cells without cytotoxicity and did not hinder cell proliferation. The MUA-AuNP showed suitable resolution for in vivo study in mouse models using visible light fluorescence and X-ray microscopy by tracing the nanoparticle loaded tumor cells. Suarasan et al. demonstrated cellular uptake and tracking of gelatin-coated AuNPs inside ovarian cancer cells by a two-photon photoluminescence analyzed using fluorescence lifetime imaging (FLIM) [39]. Examination of the spectroscopic profile of the intrinsic signals inside cells confirmed the plasmonic property of photoluminescence emitted by AuNP, with continuous irradiation that indicated the photostability of the two-photon excited photoluminescence (Figure 4).

### 4.3. Dark Field Microscopy

Dark field microscopy is a type of optical microscopy that utilizes the darkfield illumination technique, which removes dispersed light so that only the scattered beams hit the sample enabling clear imaging of unstained and transparent tissue specimens. In particular, darkfield microscopy shows suitable performance when the refractive indices of the sample are very close to those of its surroundings, such as in small aquatic organisms and cells, which are hardly detectable with conventional brightfield microscopy. AuNPs scatter strongly on LSPR frequency so that treated specimens can be visualized as bright spots with the characteristic plasmonic color of dark field microscopy [127]. Moreover, owing to the high resistance to photobleaching, AuNP is widely applied as long-time observation imaging probe for biological samples with high resolution and sensitivity [128]. Patskovsky et al. used PEGylated AuNPs that specifically targeted CD44-expressing cancer cells [40]. Simply, anti-human CD44 monoclonal antibodies were tethered to AuNPs using poly(ethylene glycol) (PEG) linkers via covalent binding with sulfur and gold. The prepared probes exhibited low phototoxicity and enabled three-dimensional (3D) darkfield imaging with localization and spectroscopic identification of fixed CD44-expressing cancer cells. Gong et al. proposed AuNPs as plasmon scattering probes for darkfield imaging of live cancer cells [41]. Gold nanospheres and nanorods were conjugated with anti-EGFR antibodies in an aqueous solution and delivered into an oral squamous cell carcinoma cell line by receptor-mediated endocytosis. Darkfield microscopy indicated that the AuNPs could be provided as different plasmon scattering probes by varying the size and formulation, with a 100 nm spectral separation in the absorption band (Figure 4).

### 4.4. Optical Coherence Tomography (OCT)

Optical coherence tomography (OCT) is a high-resolution imaging diagnostic technique capable of imaging microscopic structures inside biological tissues by combining light interference and confocal microscopy principles. By measuring light in the NIR and far-IR regions, the light reflected inside the tissue is assessed. OCT enables real-time subcellular imaging with high resolution and 1–2 mm depth profiling. AuNPs can enhance the resolution and detection limits of OCT owing to their extraordinary optical properties. AuNPs of specific formulation such as nanorods or nanoshells have superior absorption properties in the NIR and far-IR regions, which are the frequently used wavelengths in OCT. Moreover, the LSPR of AuNPs can magnify the scattering property of tissue specimen, and therefore highly improve the resolution of in situ OCT imaging [129]. Jie et al. applied gold nanoshells as a contrast agent for cardiovascular cell imaging in a catheter-OCT combined method [42]. Gold nanoshells led to substantial enhancement in the backscattered signal from the individual cells, which was attributed to the large backscattering cross-section of gold nanoshells in OCT laser wavelength. OCT images showed that subpopulations of cells could be clearly identified by incorporating the gold nanoshells. Moreover, the photothermal properties of AuNPs could be combined with OCT (PT-OCT) to enable simultaneous cancer targeting and photothermal therapy. Peng et al. synthesized pH-sensitive AuNPs, which aggregated under the low pH conditions in cancer cells [43]. The PT-OCT system distinguished signal differences between cancer cells and normal cells by detecting optical path length variations in the cancer cells, which did not change much in normal cells. The PT-OCT test was performed with an OCT light source and a laser wavelength for photothermal excitation, suggesting applicability in highly sensitive cancer theragnosis system (Figure 4).

### 4.5. Photoacoustic Imaging (PAI)

**Figure 5:**
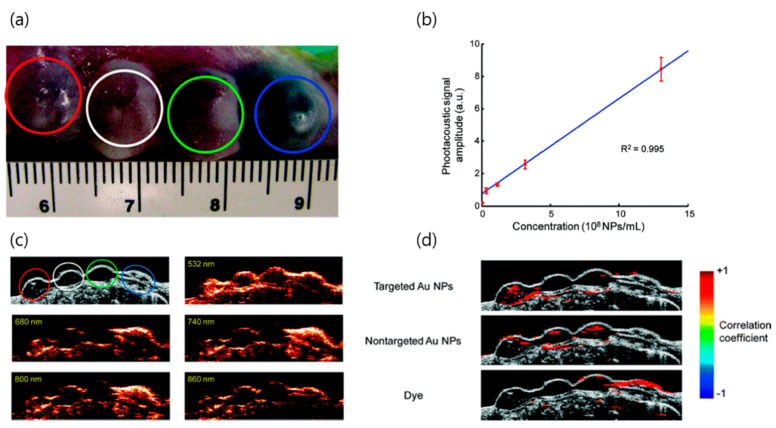
Multiwavelength PAI to tumor-mimicking gelatin implants on ex vivo mouse tissue. (**a**) Photograph of the gelatin implants in mouse tissue. Red, white, green, and blue circles indicate cells with targeted AuNPs, control A431 cells, A431 cells with mPEG-SH coated AuNPs, and NIR dye, respectively. Schematic diagram represents the gelatin two-layer model laden with cells; (**b**) Photoacoustic signal amplitude changes according to the AuNP concentration; (**c**) Ultrasound and photoacoustic images of gelatin implants at different laser wavelength. Implants with antibody labeled AuNPs (red circle) exhibited greater photoacoustic signals than the other implants; (**d**) Correlation coefficient measured by merged ultrasound and photoacoustic images. Measured correlation coefficient was high in implants with antibody labeled AuNPs. Reproduced from [46], copyright permission by ACS Publication 2009.

Photoacoustic imaging (PAI) is a real-time imaging tool capable of analyzing the anatomical structure and molecular quality of tissues based on energy conversion from light to sound. Since PAI uses an ultrasonic signal, there is less scattering inside the tissue than with light, and a high spatial resolution (5 µm) and deeper depth penetration (5–6 cm) are achieved [130]. In particular, through the use of an exogenous contrast agent, resolution can be significantly increased to observe subcellular images and molecular events. Owing to AuNP’s strong and controllable absorption generated by LSRP, it has been actively researched as a contrast agent for PAI. Manivasagan et al. demonstrated a doxorubicin loaded fucoidan-capped AuNPs (DOX-Fu AuNPs) for a multimodal system with drug delivery and PAI. [44] Fucoidan was used as the capping and reducing agent of AuNPs, prior to conjugating the DOX. DOX-Fu-AuNPs used as a PAI contrast agent for noninvasive detection of MDA-MB-231 cells exhibited highly enhanced photoacoustic signals owing to the optical scattering inside the cells. Several strategies have been implemented to enhance the resolution of PAI. Yijing et al. reported folding of AuNP strings into plasmonic vesicles to enhance PAI [45]. The hollow plasmonic vesicles containing string of AuNPs were synthesized using a stepwise self-assembly method. The prepared probes presented advantages of tailored optical and physical properties which were achieved by controlling the spatial arrangement of the AuNPs. The results indicated that the prepared vesicles showed strong absorption in the NIR region owing to the presence of the AuNP string that led to highly efficient PAI. Cancer cell receptor targeting by antibody conjugation is a novel strategy for cancer selective detection with PAI. Mallidi et al. conjugated anti-epidermal growth factor receptor (EGFR) antibody to AuNPs [46]. Upon binding to the cell surface, AuNPs underwent molecule-specific aggregation and their plasmon resonance frequency exhibited a red shift. The results of this PAI showed high selectivity and sensitivity to tumor-mimicking gelatin implants in an ex vivo mouse tissue (Figure 4 and Figure 5).

## 5. Conclusions and Future Perspectives

AuNP is one of the most attractive materials in the field of developing cancer theragnosis owing to its extraordinary physicochemical properties that provide several clinical benefits. This review has summarized recent progress of AuNPs in the field of photothermal treatment, photodynamic treatment, and cancer imaging, such as SERS, multiphoton photoluminescence imaging, darkfield microscopy, OCT, and PAI. Biocompatibility and physicochemical characteristics, based on the formulation and size of AuNPs, for consideration in the development of AuNP-based cancer theragnostic systems have been discussed. AuNPs combine the advantages of tailorable absorbance and scattering properties, with specific physicochemical properties, such as EPR, LSPR, SERS, and photothermal effects, which can be potentially applied in several cancer theragnostic probes. Moreover, from the findings described herein, solubility, stability, biofunctionality, cancer targetability, and biocompatibility of AuNPs can be significantly enhanced through various engineering methods, such as conjugation with polymers, surface coating, or incorporation of other nanomaterials. In conclusion, the results presented in this review support the promising prospect of using AuNP-based probes for improved cancer theragnosis. Although AuNP-based theragnosis has some limitations to be overcome by future research, we expect further developments to provide innovative results and improve its clinical potential for novel cancer therapies. 

## Figures and Tables

**Table 1:** Classification of recent research on the use of gold nanoparticles (AuNPs) in cancer theragnosis and their characteristics, formulation, modification, and experimental methods.

Classification	Theragnostic Modules	Required Characteristics	NP Formulation	NP Modification	Experimental Methods	Reference
Phototherapy	PTT	LSPR	Rod	BSA, Macrophage	In vivo	[14]
		LSPR	Rod	None	In vitro and In vivo	[15]
		TPA	Rod	Polypyrrole coating	In vitro	[16]
		LSPR/EPR effect	Rod	None	In vivo	[17]
		LSPR	Shell	Anti-HER2 antibody, silica	In vitro	[18]
		LSPR	Shell	T cell	In vivo	[19]
		LSPR	Flowers	γFe2o2	In vivo	[20]
		LSPR	Flowers	None	In vitro and In vivo	[21]
		LSPR	Ring	Antiibody	In vitro	[22]
	PDT	EPR effect	Sphere	C11Pc	In vitro and In vivo	[23]
		EPR effect	Sphere	Antibody, C11Pc, PEG	In vitro and In vivo	[24]
		EPR effect	Sphere	Protoporphyrin IX	In vitro	[25]
		EPR effect	Sphere	Hematoporphyrin	In vitro	[26]
		LSPR	Sphere	Protoporphyrin IX	In vitro	[27]
		EPR effect	Sphere	5-aminolevulinic acid	In vitro	[28]
		LSPR/EPR effect	Rod	Indocyanine green	In vitro	[29]
	PTT & PDT	LSPR	Cages	Hypocrellin	In vitro	[30]
		LSPR	Stars	Chlorin e6	In vivo	[31]
		LSPR/EPR effect	Rod	Indocyanine green	In vitro	[32]
		LSPR/EPR effect	Rod	Cetyltrimethylammonium bromide, PEG	In vivo	[33]
		LSPR	Rod	Hematoporphyrin, Silica	In vivo	[34]
Imaging	SERS	LSPR/SERS	Sphere	Anti-CD3/CD19 antibody, MBA, PEG	In vitro	[35]
		LSPR/SERS	Sphere	Mn, porphyrin-phospholipid	In vitro	[36]
		LSPR/SERS	Sphere	4-ATP attachment	ex vivo	[37]
	Multiphoton photoluminescence	Photoluminescence	Sphere	MUA	In vitro and In vivo	[38]
		Photoluminescence	Sphere, rod, triangle	Gelatin coating	In vitro	[39]
	Dark field microscopy	LSPR	Sphere	Anti-CD44 antibody, PEG	In vitro	[40]
		LSPR	Sphere, rod	Anti-EGFR antibody, PEG	In vitro	[41]
	OCT	Absorption property, LSPR	Shell	None	In vivo	[42]
		Photothermal property, absorption property, LSPR	Sphere	Hydrolysis-susceptible Citraconic amide	In vitro	[43]
	PAI	LSPR, PA	Sphere	DOX loading, Fu capping	In vitro	[44]
		LSPR, PA	Sphere string	BCP tethering	In vivo	[45]
		LSPR, PA	Sphere	Anti-EGFR antibody	ex vivo	[46]
